# What Do We Need to Know to Improve Diagnostic Testing Methods for the 2019 Novel Coronavirus?

**DOI:** 10.7759/cureus.8263

**Published:** 2020-05-24

**Authors:** Zahid Mustafa, Masoumeh Ghaffari

**Affiliations:** 1 Internal Medicine, School of Medicine, University of California, Riverside, USA; 2 Internal Medicine, School of Medicine, University of California/Riverside Community Hospital, Riverside, USA

**Keywords:** 2019 novel coronavirus, sars-cov-2, covid-19, coronavirus, diagnostic testing, testing methods

## Abstract

There is widespread agreement that reliable, fast, and easy-to-produce diagnostic testing methods that have high sensitivity and specificity are essential for guiding appropriate responses to the severe acute respiratory syndrome coronavirus 2 (SARS-CoV-2) outbreak. At the present time, there are important unanswered questions about testing methods for SARS-CoV-2. This review article interprets recent findings related to the principal testing methods used to diagnose SARS-CoV-2, including reverse-transcription polymerase chain reaction (RT-PCR), chest imaging, and immunoassay. We discuss the value and limitations of these approaches and suggest directions for future research that can advance the understanding of diagnostic methods. Addressing areas of uncertainty will improve clinical outcomes and allow more effective policies to be implemented to control the disease.

## Introduction and background

The novel coronavirus that emerged in December 2019, severe acute respiratory syndrome coronavirus 2 (SARS-CoV-2), has spread rapidly around the world. Medical systems have come under severe stress as a result, presenting serious clinical and public health management challenges. There is widespread agreement that reliable, fast, and easy-to-produce diagnostic testing methods that have high sensitivity and specificity are essential for guiding appropriate responses to the outbreak [1]. This literature review article interprets recent findings related to testing methods and discusses the value and limitations of existing approaches. We also suggest directions for future research that can improve the understanding of diagnostic methods. At the present time, there are important unanswered questions about testing methods for SARS-CoV-2. Confronting areas of uncertainty will improve diagnostic testing methods, allowing more effective policies to be implemented to battle the disease.

## Review

Reverse-transcription polymerase chain reaction (RT-PCR)

The most widely used method for detecting new SARS-CoV-2 infections in the United States is reverse-transcription polymerase chain reaction (RT-PCR) [2]. Most RT-PCR methods for detecting SARS-CoV-2 use a fluorescent deoxyribonucleic acid (DNA) probe that binds to a specific SARS-CoV-2 target gene. If a patient sample contains SARS-CoV-2, the DNA probe amplifies the target gene. During the amplification process, a fluorescent signal is emitted that can be detected and characterized, establishing the presence of SARS-CoV-2. Laboratory experiments show that RT-PCR has high sensitivity and specificity when identifying in-vitro transcribed RNA that matches the sequence of SARS-CoV-2 [3]. However, there is reason to believe that the excellent performance RT-PCR achieves in carefully designed laboratory experiments is not being achieved in tests administered to the public. Case reports describe patients who have tested negative for SARS-CoV-2 using RT-PCR who are later confirmed to be infected [4-5]. Multiple studies of SARS-CoV-2 conducted in China using chest computed tomography (CT) scans discuss known and likely positive cases that have negative RT-PCR results [6-8]. These studies report RT-PCR sensitivities between 71% and 88%. If these findings are representative of clinical RT-PCR sensitivity, a significant percentage of individuals with coronavirus disease 2019 (COVID-19) are being incorrectly diagnosed as false negatives, infected patients who are classified as disease-free.

The discrepancy between laboratory and clinical performance raises two important issues about the use of RT-PCR to detect SARS-CoV-2 that should be addressed. The first is obtaining reliable estimates of the sensitivity and specificity of RT-PCR tests used to detect COVID-19 in individuals. Having a better understanding of the sensitivity and specificity of RT-PCR will help diagnose and treat both the healthy and the sick. If sensitivity is low, symptomatic individuals will need to be tested repeatedly so that infected individuals are not incorrectly diagnosed as disease-free. Low sensitivity also implies treating all individuals with symptoms of SARS-CoV-2 and quarantining anyone who appears to be sick or has had exposure to a person diagnosed as infected. The reason is that when a test has low sensitivity, negative test results provide little additional information about the probability that an individual is infected, making it harder to rule out the possibility of an individual having the disease. Knowing RT-PCR sensitivities and specificities can also improve epidemiological models. Existing models do not include estimates of RT-PCR performance as relevant variables. Incorporating sensitivity and specificity into epidemiological models will help researchers better predict the spread of the disease, examine how policy interventions affect transmission rates, and determine the resources needed to minimize health and economic damage.

The second issue involves characterizing the sources of variation in RT-PCR sensitivity and specificity reported in the clinical literature. The most obvious possible cause of variation is the fact that different tests use different protocols to establish the presence or absence of SARS-CoV-2. The procedure endorsed by the World Health Organization (WHO) recommends first screening for the presence of the envelope protein gene (E) and then confirming it by testing for the RNA-dependent RNA polymerase gene (RdRp) [3]. The Chinese Center for Disease Control and Prevention (CDC) assay targets the nucleocapsid protein gene (N) and the open reading frame 1b gene (Orf1b). The United States Centers for Disease Control and Prevention protocol uses two probes for the N gene and one probe for the RNase P gene (RP) to provide a diagnosis. Academic and private labs continue to develop and deploy their own proprietary RT-PCR systems, facilitated by the decision of the United States Food and Drug Administration (FDA) on February 29, 2020, to permit certain university and corporate labs to process tests without obtaining prior approval from the FDA. Studies should be conducted to compare the sensitivity and specificity of the different RT-PCR test platforms to determine which systems perform best and why.

Another potential source of variation in RT-PCR sensitivity and specificity for SARS-CoV-2 is differences in sample collection. RT-PCR performance may depend on the type of sample and how it is collected. Many different sampling methods are being used: swabbing the nasopharyngeal region, swabbing the oropharyngeal region, obtaining sputum, aspirating the nasopharyngeal region, conducting a bronchoscopy, and collecting saliva, stool, or urine for analysis. A study of seven family members from China reports that RT-PCR results changed depending on the type of sample collected [9]. One possible explanation for this finding is that viral loads differ in different types of samples. As yet, there is no consensus on where viral loads are highest in the body and, hence, where it is easiest to detect the presence of the virus. Initial studies based on small sample sizes describe the nose of the upper respiratory tract and the lower respiratory tract as yielding the greatest viral loads [9-11]. More recent published work based on larger sample sizes indicates that SARS-CoV-2 nucleic acid is most easily detected in alveolar lavage fluid, followed by sputum, and then in nasal and pharyngeal swabs [12-14]. Further studies that apply multiple sampling techniques to a given individual should be conducted to confirm the results of recent work and inform clinical sampling methods.

Time is another factor that likely contributes to variation in the sensitivity and specificity of SARS-CoV-2 RT-PCR. How long it takes after initial infection for an individual to test positive and how long it takes after a patient is no longer symptomatic to return a negative test result are significant unknowns that vary between people and may be a function of whether a person is immunocompromised or is taking antiviral medication. Samples should be collected from the same individuals at consistent intervals to determine how the sensitivity and specificity of a particular sampling method vary over time and to help understand the sources of variation in sensitivity and specificity between patients. One troubling possibility for individual health and slowing the spread of the disease is that low viral loads at the onset of infection may account for a large share of false-negative RT-PCR results. This hypothesis should be investigated in future studies. The question of the relationship between symptom onset, viral loads, and RT-PCR test results is closely related to the issue of asymptomatic carriers. Do asymptomatic carriers differ in how their bodies respond to SARS-CoV-2? Or do these individuals’ healthy appearances reflect low viral loads as compared to patients who display symptoms? A study that analyzed 17 symptomatic patients and one confirmed asymptomatic patient in China suggests the former hypothesis [10]. Further research on the limits of detection using RT-PCR is needed to characterize definitively what makes asymptomatic carriers different from the sick.

Chest imaging

Imaging technologies, such as chest CT scans and ultrasound, have been proposed as tools to diagnose COVID-19. Chest CT scans can identify cases of viral pneumonia, even though RT-PCR results for SARS-CoV-2 at the time images are taken are negative [7]. A retrospective study of 1,014 patients in Wuhan, China, reports that chest CT has a sensitivity of 97% and may be considered as a primary detection tool because it is a more reliable, practical, and rapid method than RT-PCR [15]. Another study of 1,099 confirmed cases throughout China finds that chest CT scans are 86% sensitive [16]. Studies should be performed to determine the conditions under which chest CT scans should be used as a tool to complement RT-PCR analysis. Symptomatic patients and asymptomatic individuals who may have been exposed to SARS-CoV-2 should be given chest CT scans and RT-PCR and the results of these diagnostic methods compared. Including asymptomatic patients in the sample is necessary to avoid biasing results. Although most RT-PCR positive asymptomatic individuals show peripheral opacities on chest CT scans, a small percentage do not [17-18]. One possibility is that this discrepancy reflects differences in disease severity. If so, chest CT scans would be a useful tool for evaluating the severity of the infection.

Ultrasound has also been examined in a case report and a study of 20 COVID-19 patients as a possible technique for facilitating the diagnosis of SARS-CoV-2 [19-20]. An advantage of ultrasound as compared to chest CT scans is that ultrasound does not emit harmful radiation. Therefore, it is possible to periodically administer ultrasound, allowing for closer monitoring of a patient’s condition. This benefit must be balanced against the risk of infection that persistent monitoring of patients suspected to have SARS-CoV-2 creates for ultrasound technicians and physicians. The tradeoffs involved with using ultrasound as a substitute for chest CT scans in diagnosing COVID-19 should continue to be explored.

Immunoassay

Immunoassays detect SARS-CoV-2 antigens and the antibodies the body produces in response to infection. Immunoassays that detect the presence of SARS-CoV-2 antigens generally work by binding SARS-CoV-2 antibodies that are specific to a SARS-CoV-2 antigen to a membrane. A patient sample is added to a well that contains a reporter molecule-linked antibody that is also specific to the SARS-CoV-2 antigen. If the patient sample contains a SARS-CoV-2 antigen, the SARS-CoV-2 antigen binds to the SARS-CoV-2 antibody on the membrane, concomitantly binding the reporter molecule-linked antibody. Immunoassays that detect the SARS-CoV-2 antibodies the body produces generally work by binding a synthetically produced SARS-CoV-2 antigen to a well. A patient plasma sample is added to the well. If the patient’s plasma contains SARS-CoV-2 antibodies, these antibodies will bind to the synthetically made SARS-CoV-2 antigen. Another antibody linked to a reporter molecule that is specific for the SARS-CoV-2 antibody is then added to the well. The reporter molecule-linked antibody for both types of immunoassays can be analyzed through fluorescence or a color change, indicating the presence or absence of SARS-CoV-2. These tests have the potential to provide information about current and past SARS-CoV-2 infections, giving them diagnostic and epidemiological value. At the time of writing, there is no antigen detection test that has been broadly deployed. Such a test would theoretically be able to provide point-of-care results, yielding immediate data for treatment and disease control. However, experience with influenza viruses has shown that antigen detection tests have poor sensitivity [21]. A number of antigen detection tests are under development by private manufacturers. These tests should be validated in peer-reviewed studies when they are brought to the market to determine their usefulness.

Antibody detection assays have also been proposed to detect infection by SARS-CoV-2. A research team in China created a test to detect the presence of immunoglobulin M (IgM) and immunoglobulin G (IgG) antibodies for SARS-CoV-2 in serum. Sixteen patients known to be infected were tested using the assay. Initial IgM and IgG antibody titers were low or below the limit of detection. After five days, IgM sensitivity was 50% and IgG sensitivity was 81%. After 10 days, IgM sensitivity increased to 81% and IgG sensitivity increased to 100% [22]. Another antibody detection test developed in China provides results within 15 minutes. The test has a sensitivity of 89% and a specificity of 91%, and the authors report that several Chinese companies are creating similar products [23]. Further studies should be done to validate the performance of these antibody assays. An important question that will need to be addressed is how individuals differ in their development of antibodies. One experiment that used plasma from 82 confirmed and 58 probable cases of COVID-19 found that 22% of confirmed cases had negative IgM test results. The authors note that some of these cases were likely sampled before the individuals produced a sufficient antibody response to be detected. But two infected patients with severe symptoms had a negative antibody test approximately three weeks after the onset of symptoms [24]. Determining how individuals differ in their antibody response to SARS-CoV-2 is a prerequisite to using these types of immunoassay techniques to diagnose patients and to knowing whether individuals have been unknowingly infected in the past.

## Conclusions

Accurate, reliable, and fast diagnostic methods for SARS-CoV-2 are needed to inform clinical and public health decisions. Significant uncertainties about testing methods exist: about their performance, about what causes differences in sensitivity and specificity, about the ability of different approaches to complement each other, and about whether they can be validated. Answering these questions over the coming weeks and months will improve testing, increasing confidence in our ability to detect COVID-19 infections, and allowing better policies to be crafted to address the unfolding crisis.

## References

[1] Sharfstein JM, Becker SJ, Mello MM (2020). Diagnostic testing for the novel coronavirus. JAMA.

[2] Udugama B, Kadhiresan P, Kozlowski HN (2020). Diagnosing COVID-19: the disease and tools for detection. ACS Nano.

[3] Corman VM, Landt O, Kaiser M (2020). Detection of 2019 novel coronavirus (2019-nCoV) by real-time RT-PCR. Euro Surveill.

[4] Winichakoon P, Chaiwarith R, Liwsrisakun C, Salee P, Goonna A, Limsukon A, Kaewpoowat Q (2020). Negative nasopharyngeal and oropharyngeal swab does not rule out COVID-19. J Clin Microbiol.

[5] Huang P, Liu T, Huang L (2020). Use of chest CT in combination with negative RT-PCR assay for the 2019 novel coronavirus but high clinical suspicion. Radiology.

[6] Fang Y, Zhang H, Xie J, Lin M, Ying L, Pang P, Ji W (2020). Sensitivity of chest CT for COVID-19: comparison to RT-PCR. Radiology.

[7] Xie X, Zhong Z, Zhao W, Zheng C, Wang F, Liu J (2020). Chest CT for typical 2019-nCoV pneumonia: relationship to negative RT-PCR testing. Radiology.

[8] Bernheim A, Mei X, Huang M (2020). Chest CT findings in coronavirus disease-19 (COVID- 19): relationship to duration of infection. Radiology.

[9] Chan JF, Yuan S, Kok KH (2020). A familial cluster of pneumonia associated with the 2019 novel coronavirus indicating person-to-person transmission: a study of a family cluster. Lancet.

[10] Zou L, Ruan F, Huang M (2020). SARS-CoV-2 viral load in upper respiratory specimens of infected patients. N Eng J Med.

[11] Pan Y, Zhang D, Yang P (2020). Viral load of SARS-CoV-2 in clinical samples. Lancet Infect Dis.

[12] Liu R, Han H, Liu F (2020). Positive rate of RT-PCR detection of SARS-CoV-2 infection in 4880 cases from one hospital in Wuhan, China, from Jan to Feb 2020. Clin Chim Acta.

[13] Yu F, Yan L, Wang N (2020). Quantitative detection and viral load analysis of SARS-CoV-2 in infected patients. Clin Infect Dis.

[14] Wang H, Li X, Li T, Zhang S, Wang L, Wu X, Liu J (2020). The genetic sequence, origin, and diagnosis of SARS-CoV-2. Eur J Clin Microbiol Infect Dis.

[15] Ai T, Yang Z, Hou H (2020). Correlation of chest CT and RT-PCR testing in coronavirus disease 2019 (COVID-19) in China: a report of 1014 cases. Radiology.

[16] Guan WJ, Ni ZY, Hu Y (2020). Clinical characteristics of coronavirus disease 2019 in China. N Eng J Med.

[17] Lin C, Ding Y, Xie B, Sun Z, Li X, Chen Z, Niu M (2020). Asymptomatic novel coronavirus pneumonia patient outside Wuhan: the value of CT images in the course of the disease. Clin Imaging.

[18] Ling Z, Xu X, Gan Q, Zhang L, Luo L, Tang X, Liu J (2020). Asymptomatic SARS-CoV-2 infected patients with persistent negative CT findings. Eur J Radiol.

[19] Buonsenso D, Piano A, Raffaelli F, Bonadia N, de Gaetano Donati K, Franceschi F (2020). Point-of-care lung ultrasound findings in novel coronavirus disease-19 pnemoniae: a case report and potential applications during COVID-19 outbreak. Eur Rev Med Pharmacol Sci.

[20] Peng QY, Wang XT, Zhang LN, Chinese Critical Care Ultrasound Study G (2020). Findings of lung ultrasonography of novel corona virus pneumonia during the 2019-2020 epidemic. Intensive Care Med.

[21] Loeffelholz MJ, Tang YW (2020). Laboratory diagnosis of emerging human coronavirus infections - the state of the art. Emerg Microbes Infect.

[22] Zhang W, Du RH, Li B (2020). Molecular and serological investigation of 2019-nCoV infected patients: implication of multiple shedding routes. Emerg Microbes Infect.

[23] Li Z, Yi Y, Luo X (2020). Development and clinical application of a rapid IgM-IgG combined antibody test for SARS-CoV-2 infection diagnosis. J Med Virol.

[24] Guo L, Ren L, Yang S (2020). Profiling early humoral response to diagnose novel coronavirus disease (COVID-19). Clin Infect Dis.

